# Academic achievement: the effect of project-based online learning method and student engagement

**DOI:** 10.1016/j.heliyon.2022.e11509

**Published:** 2022-11-12

**Authors:** Zelhendri Zen, Reflianto, Syamsuar, Farida Ariani

**Affiliations:** a Educational Technology Department, Faculty of Education, Universitas Negeri Padang, 25171, Air Tawar Barat, Padang, Indonesia; b Management Department, Faculty of Economic, Universitas Sumatera Barat, 25523, Kampung Gadang, Pariaman, Indonesia; c Physic Education Department, Faculty of Sport Sciences, Universitas Negeri Padang, 25171, Air Tawar Barat, Padang, Indonesia; d Management Department, Faculty of Economic, Universitas Sumatera Barat & IAI Sumbar, Pariaman, Indonesia

**Keywords:** Project-based online learning, Student engagement, Academic achievement

## Abstract

The commitment of higher education to produce job-ready and highly competitive graduates requires that it endow graduates with broad knowledge and innovative technologies, including business skills facing various challenges of project-based digital learning for future achievement. The study aimed to analyze the effect of Project-Based Online Learning (PBOL) and student engagement on academic achievement. The research method used a mixed-method convergent parallel design. Qualitative data collection is through interviews, observational and documentation forms, and quantitative data is through questionnaires and portfolios. A qualitative data interpretation used content analysis, while quantitative data used the Two-Way ANOVA test. The results showed that students' perceptions of implementing the PBOL method and student engagement increased their academic performance to become new entrepreneurs through the experience they gained during project-based learning. This method could also create a fun learning atmosphere that affects student engagement behavior. The research contributes an alternative method for entrepreneurship lecturers to enhance their educational performance and success.

## 1 Introduction

The not yet optimal quality of learning during the COVID-19 pandemic has caused a decrease in student involvement in learning (Lambert and Rennie, 2021). This condition causes the phenomenon of learning loss that worries the world of education (Dorn et al., 2020). To overcome this, lecturers should apply the digital-based learning methods needed in the new-normal era (Reflianto et al., 2021). One is to employ learning methods that could increase engagement, creativity, and technology mastery through project-based, independent learning experiences.

The method is Project Based Online Learning (PBOL), which leads to strengthening entrepreneurial insight and skills. Boss and Krauss (2014) stated that mastery of technology and broad insight into the business world could encourage students' interest in involving themselves in becoming new entrepreneurs. The PBOL method has various advantages over other methods, especially making it easier for lecturers and students to communicate both inside and outside the classroom and for students to access lecture materials through the Microsoft Teams platform and social media.

They integrated digital classroom facilities on these two platforms into a ubiquitous learning approach using laptops, tablets, and smartphones (Suartama et al., 2020). These digital classroom tools will allow students to acquire distinct learning experiences based on their needs (Virtanen et al., 2018). Bryer and Zavattaro (2011) stated that the affordability of social media strongly influenced the success of digital-based learning.

Currently, most of the existing research is still in traditional PBL research, is nothing research on PBOL. Pan et al. (2019) showed that the PBL method contributes positively to increasing the motivation of students to take part in the learning process. Raza et al. (2019) reported that engagement and motivation affect the academic achievement of management students at the Karachi University of Pakistan using the PBL method. The two studies above only focus on the application of traditional PBL methods. Unlike this research, it focuses more on applying PBOL's integrated digital approach to implementing the independent learning program. It is a novelty of this research, a study, and an analysis of the PBOL method and social media effectiveness in entrepreneurship education in universities. Verdugo and Villarroel (2021) revealed that social media could strengthen higher education in sustainable entrepreneurship because they can incorporate these channels as a formal source of information for learning.

The application of this method is to build collaboration and communication between lecturers and students through text, images, and videos. Arias et al. (2018) said strengthening entrepreneurship learning digital services to students is one goal of improving the quality of learning in higher education, especially in entrepreneurial learning that demands high competence of lecturers and students in seeing the prospective market and business opportunities in the future. Implementing a well-planned PBOL approach will improve the quality of graduates in responding to global business challenges, including the ASEAN Economic Community (AEC).

An important task of higher education is to educate students entering the future labor market as it increases their competitiveness and promotes the long-term development of society (Crosling et al., 2015).

Research by Martín et al. (2017) has suggested the need to encourage students to innovate by supporting their autonomy during learning tasks. The project-based learning is based on active construction learning (Shin et al., 2021), emphasizing knowledge construction. Creating new knowledge allows students to test and achieve their ideas in the way they want and promotes innovation competence. So, The PBOL method is indispensable to encouraging lecturers in higher education to adopt project-based online learning to increase student engagement and academic achievement.

To guide the research, the research questions are divided into five: (1) How is the effectiveness of the PBOL method in supporting the entrepreneurial learning process? (2) How is student engagement in entrepreneurship education using the PBOL method? (3) Is there a difference between the PBOL and the conventional method of academic achievement? (4) Is there a difference between high, medium, and low student engagement in academic achievement? (5) How is the interaction between the method and student engagement in academic achievement?

## 2 Literature review

### 2.1 Project-based learning

Project-based learning, or PBL, is a learning method that focuses students on the complex problems needed to investigate and understand lessons through inquiry. This method also aims to guide students in collaborative projects that integrate various subjects, which provide opportunities for students to explore content in several ways that are meaningful to themselves and conduct collaborative experiments (Belwal et al., 2020).

Lokey-Vega and Bondeson (2017) said project-based online learning or PBOL is a learning method that uses problems as a first step in gathering and integrating new knowledge based on real-time experiences and activities. The PBOL guides students in solving complex problems that must be researched and understood. Then Hogue and Kapralos (2011) revealed that the PBOL method is a distance learning method using digital media to present learning to students. Using this method, the teacher guides students to identify strategies to overcome the problems they face by analyzing complex problems according to their interests and talents.

Carnawi et al. (2017) also said that project-based learning is a learning method that uses projects as a learning tool to achieve competency attitudes towards mastery of concepts, creativity, leadership, knowledge, and the courage to take risks and skills. This learning is a substitute for teaching that is always speaker-centric. The emphasis is on student activities that can generate meaningful outputs. The principle of the project-based learning method is to emphasize students' skills in overcoming problems experienced in real life. This research can produce an alternative learning method needed the educators during COVID-19. Under the student abilities, students can solve problems, find concepts, and build principles of handling problem management correctly, which can become new knowledge they acquire directly from meaningful learning experiences. PBOL is the development of a PBL method that integrates online learning.

The seven core components of this learning method are: attracting students' interest and attention by asking students relevant questions related to case studies, explaining student assignments through video tutorials, conducting formative assessments to measure student understanding, building content knowledge about project-based learning, including readings, videos, and pictures to make them understand and remember to complete their projects. It also provides tutorials that can help students understand project work. Tutorials explain how to do the task, grade rubrics, and samples that students can observe through videos and allow students to think critically in completing the project assignments (Lokey-Vega and Bondeson, 2017).

### 2.2 Strengthening entrepreneurship through technology-based learning

The active penetration of information and communication technology into the ever-growing education system, followed by the increasing number of educational resources on the internet, urges educators to rethink how the education process during the COVID-19 pandemic can continue. Based on Carnawi et al. (2017), new technology is a core need for the intellectual development of students and socio-economic society, adhering to the basis of production, transmission, and assimilation of knowledge. This context demands systems and learning methods that enable universities to prepare graduates for good jobs in the future.

In a globalized world economy, Samuel and Rahman (2018) stated that the concept of priority economic development is one development of productive entrepreneurial businesses. They encourage productivity by creating broad business jobs to open an independent creative economy business. The economic concept attracts national and foreign investors to increase their business through the global market. During the Industrial Revolution 4.0, learning activities no longer focused on face-to-face classroom activities, but about 40–60 percent of education services provided online learning preference. By implementing digital-based distance learning with social media, websites, and others, Cooper (2010) revealed that learning services like this could overcome the problem of limited face-to-face access in schools. To prepare for this online-based distance class, Sayan (2016) suggests that lecturers apply various digital media, such as WhatsApp, Facebook, Instagram, or web-based media, such as Google Classroom, Google Meet, Edmodo, and Microsoft Teams, and others.

### 2.3 Student engagement

Student engagement is knowing how effectively the student learning process is during learning (Kahu, 2013). Engagement arises because students participate in educational scenarios where they explore their potential knowledge and insights (Attard and Holmes, 2020). Three elements of student engagement concepts in the recent literature on the definition of student engagement are behavioral engagement, emotional engagement, and cognitive engagement (Phothongsunan, 2020).

Skinner and Pitzer (2018) said that the engagement of student behavior demonstrates a positive impact on them when participating in all learning activities and complying with established norms, such as students having to complete their assignments on time. Emotional engagement refers to students' emotional reactions at school. It relates to student interests such as boredom, happiness, sadness, or anxiety. The more students are happy with the subject, the more active and take part in the learning. Cognitive engagement is a specific engagement associated with a psychological investment.

## 3 Method

### 3.1 Research purposes

This study aimed to analyze the applying the PBOL method's effect on student engagement and academic achievement in entrepreneurship courses. An in-depth analysis would analyze the effectiveness of PBOL and student engagement and examined the effect and interaction of the application of PBOL methods and student engagement in improving entrepreneurial learning outcomes in the new-normal era.

### 3.2 Research design

This research used the mixed method convergent parallel design (Creswell and Clark, 2017). There were two stages of implementing data collection in the field. First, collect qualitative and quantitative data at relatively the same time. Collecting qualitative data through interviews with participants and quantitative data, this research used a quasi-experimental approach to measure differences in the effectiveness of the PBOL method compared to the traditional method. Creswell W (2014) stated that the design of a quasi-experimental design provides an opportunity to investigate the effect of two treatment variables called factors on the sample group under study simultaneously.

### 3.3 Research sample

We conducted this research on fifth-semester students at the Faculty of Education, Padang State University, with four entrepreneurship lecturers and 153 students aged 20–22 as respondents. The lecturer's coding was L1, L2, L3, and L4, and the eight student respondents were S1, S2, S3, and so on. Tested the effectiveness of the PBOL method for this research using a quasi-experimental approach and divided the number of students into two classes, namely 78 for the experimental class with the PBOL method and 75 for the control class with the conventional method.

### 3.4 Research instrument

The qualitative research instrument used interview forms, observations, and documents. An interview form to collect detailed data on the responses of lecturers and students to the application of the PBOL method in entrepreneurship learning. All semi-structured interview questions were based on three main themes: behavioral engagement, emotional engagement, and cognitive engagement. A portfolio-based assessment for entrepreneurial academic achievement comprises five elements with 19 sub-indicators: 1) Concept Mastery (remembering, understanding, applying, analyzing, evaluating, and creating) Aadland and Aaboen (2020), 2 Creativity (curiosity, asking a lot, lots of ideas) (Starko, 2017), 3 Leadership (analyze and make decision, motivating, communication, responsibility) (Han et al., 2019), 4) Courage to take risks (dare to speculate, not afraid to fail, high confidence) (Rasulzada, 2017), and 5) Entrepreneurship encouragement (interest and motivation, optimism, self-confidence) (Herdjiono et al., 2017). Portfolio assessment also includes all student activities in completing routine assignments, presentations, discussion of materials and exams, and final project results taught through online learning.

All questions on the questionnaire and interview forms referred to the research theme and validated it with experts and colleagues. All interviews started with the same question: how did the lecturers conduct entrepreneurship lectures using the PBOL method during the COVID-19 pandemic? (see Appendix 1 in supplementary material). The interview stages run like a closed conversation between two professionals while still trying not to take a leading position, but being a listener and directing respondents to answer questions according to the theme. Researchers also used probing questions to verify their interpretation of the answers (Britten, 1995; Dilley, 2004). The emergence of new ideas while interviewing respondents required us to re-run the follow-up interview to complete the information. The data is said to be saturated if the information obtained is completed.

The triangulation and verification of data inter-subjectively with the evaluators to get different information to strengthen the validity of the findings (Denzin, 2020). For that reason, it used descriptive categorical analysis with a low interference level to ensure data reliability and get the data as concrete as possible (Rose and Johnson, 2020). The collection of data involved two data collectors to ensure a balance of analysis and interpretation of the findings.

The development of a questionnaire form based on previous qualitative findings and an adaptation of the experts for the student person engagement questionnaire (SESQ) (Hart et al., 2011). The research adapted qualitative findings and expert theory comprising three sub-variants: 1) behavioral engagement, 2) emotional engagement, and 3) cognitive engagement (see Appendix 2 in supplementary material). After the expert test, the content validity tests out in the field and found that the number of valid questions for the final SESQ questionnaire was 20 of 23 items. Each sub-variable includes six questions except the behavioral involvement sub-variant, which has eight questions. To ensure the reliability of the questionnaire, the researcher used Cronbach's Alpha calculation with the acquisition of 0.824.

### 3.5 Procedure and data analysis

Collecting qualitative and quantitative data in this research was conducted simultaneously. Qualitative research involved a semi-structured interview on the effectiveness outcomes and student engagement while following the PBOL method in entrepreneurship learning. The interviews were conducted directly with respondents after first asking about their willingness. Data analysis used coding to ensure the confidentiality of respondents. The interview process lasted approximately 30 min for each lecturer and student via a zoom meeting. This study recorded all interviews using smartphones and copied them to facilitate data processing. To strengthen the validity of the findings, used a triangulation evaluator (Denzin, 2020).

For reasons of reliability, used a descriptive category analysis with a low level of interference to achieve as concrete results as possible (Rose and Johnson, 2020). This research also involved two data collectors to ensure a balance of analysis and interpretation (Majid et al., 2017). While in quantitative research, the collection data used the Student Engagement in Schools Questionnaire (SESQ) with a Likert scale of one to five (e.g., one = never, five = always). The student responses were divided into three groups based on the percentage of their achievements, namely high, medium and low student involvement. Meanwhile, the academic achievement of students used the portfolio-based assessment sheets in entrepreneurship learning activities using the PBOL and conventional method for four months, supported by the use of Microsoft Teams and social media. The academic achievement of students consisted of five elements of point learning achievement based on portfolio. They were 1) Concept Mastery, 2 Creativity, 3 Leadership, 4) Courage to take risks, and 5) Entrepreneurship encouragement (see Appendix 3 in supplementary material).

The second stage was to analyze the integration of the two data findings to explain scientifically the effect of applying the PBOL method and student involvement on academic achievement in entrepreneurship education in terms of the correlation of qualitative and quantitative data that have been elaborated on comprehensively.

### 3.6 Ethical consideration

The research ethics had obtained approval from the chairman of the ethics board of the State University of Padang Dr. Syamsurizal M. Biomed. In the implementation, the researchers informed all respondents about the purpose of this research. All their answers and responses are kept confidential and anonymous by using codes and numbers for direct quotes from them. The researcher also obtained written consent from all respondents involved in this research and consciously and willingly gave written it regarding their willingness to answer questions honestly during the interview and fill out the questionnaire instrument according to the actual condition.

## 4 Results

### 4.1 The effectiveness of the PBOL method in entrepreneurship learning in the new-normal era

#### 4.1.1 Qualitative findings

The PBOL method's effectiveness shows that the data collection during online learning to use Microsoft Teams and social media can overcome the limitations of face-to-face in schools. Lecturers' success in providing understanding to students has also increased significantly. The PBOL method effectively supported distance learning, supported by digital classroom features that make it easier for lecturers and students to communicate and interact directly during learning in synchronous and asynchronous classes. It also makes it easier to provide material and facilitates access to download materials by students for independent study before entering the synchronous discussion class. Usually, lecturers give material reinforcement tasks to students a week before the synchronous class starts through the digital class feature.

The feedback process on the assignments reported by students lasted for one week by taking a two-day schedule for asynchronous classes. This asynchronous class contains question-and-answer activities regarding digital-based business project materials and their marketing that students must understand when choosing and working on their business projects. Lecturers also accompany training materials for digital-based business projects, strategies, and marketing using videos through the material upload feature in Microsoft Teams digital classes.

The learning process is fun and can stimulate students' interest and motivation in every learning activity, from completing material assignments to independent practice at home and reporting to lecturers through the Microsoft Teams digital class feature that provides upload assessment and evaluation. It is revealed from the following student statements:

Learning to use the PBOL method supported by Microsoft Teams and social media helps us to understand the digital-based business project materials, strategies, and marketing that the lecturers share with us. We can run these digital-based business projects and marketing tasks from home, according to the directions given by the lecturer. The flexible learning implementation process is not only fun but also able to build a relaxed atmosphere so that we do not feel burdened with project assignments given by lecturers. Instead, we get benefits and productive skills to create digital-based business projects after doing the projects given [Respondent S2].

The statements above describe how students' behavior and emotional atmosphere emerged following the PBOL Method in studying digital-based business projects, strategies, and marketing. Microsoft Teams and social media supported a positive impression. It appeared from their enthusiastic behavior engagement in working on assigned projects and reporting their development every week during synchronous and asynchronous classes as a follow-up to strengthening the material and evaluating the implementation of given project assignments.

In implementing entrepreneurship learning, the lecturer provides all the material according to the agreed learning schedule in the Lesson Plan. The lecturer delivers the subject in each meeting systematically, starting from mastering the material and understanding building digital-based business projects, strategies, and correct marketing to produce actual marketed products. Assignments strengthen exercises in virtual classes for students to answer several problems related to entrepreneurship material by requiring students to follow up on these problems to offer product solutions that provide great opportunities in the digital business world. Students report all assignments every week.

Understanding the material is usually as text while developing digital business projects as video recordings of independent exercises. The lecturer said that method was systematic, making it easier for students to understand and master all processes of building a digital business, from ideas, market opportunities, market management, and market strategies to market segments. They carry out assignments happily, relaxed, and free from burdens because they carry out the video tutorial instructions, as stated by some of the following students:

As students, we feel happy and relaxed without the anxiety of carrying out all the tasks given by the lecturers. We enjoyed following the digital-based business projects learning that has opportunities and a large market segment from home. We are happy to carry out training assignments for more than the hours given by the lecturer because we prefer working on the task. The systematic and gradual delivery of material also affects our acceleration in knowing, understanding, applying, analyzing, and evaluating entrepreneurship material well [Respondent S5].

The statement above shows that the success of lecturers in building a distance learning atmosphere using the PBOL method has increased student involvement in all learning activities during discussions in synchronous and asynchronous digital-based business project activities assigned to teaching materials provided by the lecturer. The PBOL method could the learning process make it easier to communicate with each other in the chatroom and make it easier to get all subjects directly from the lecturer by downloading the virtual class material from Microsoft Teams.

The availability of material that comes with video tutorials makes students enthusiastic about learning independently of their homes. When getting problems, they can communicate via WhatsApp with their lecturers directly. The willingness of lecturers to assist students in distance learning will increase active student involvement in learning, which impacts their success in carrying out project assignments given by lecturers through entrepreneurship education:

PBOL learning to use Microsoft Teams makes it easy for us to communicate via chatroom whenever and wherever students need guidance. Besides the digital class feature, using social media: WhatsApp, Facebook, and YouTube also make it easier for us to get links to materials that lecturers have uploaded. This condition has a positive impact on our involvement in participating in each learning session. We also get real-time feedback on class assignments and motivation to be actively involved in carrying out all the assigned tasks [Respondent S4].

The expressions supporting this statement got from the lecturers during interviews with them:

As lecturers, we are a little busier bcause we have to make time anytime and anywhere to serve students when they consult about their project assignments. The success of distance learning will be very successful if we can provide real-time feedback directly to students when they ask questions. Fortunately, the PBOL method using Microsoft Teams and social media makes it easier for us to communicate remotely, especially by answering students' questions and giving complete explanations with links to the materials needed. It impacts increasing students' behavioral and emotional involvement in carrying out tasks and their cognitive engagement [Respondent L1].

The statement above reveals that the complicated work and dedication of the lecturers to guide, educate, and direct students diligently is an absolute requirement for the successful implementation of this PBOL method. Because real-time lecturer feedback will affect the enthusiasm of students to carry out the tasks given to match the expected targets, active participation in learning affects their learning outcomes. In applying the PBOL method, the competence of lecturers will determine the success of their teachings like pedagogic competence, personality competence, social competence, and professional competence. Setyosar et al. (2022) revealed that teacher competence in applying the question-level and questioning strategy can motivate and encourage student engagement in self-study to enhance their skills and knowledge.

These competencies will affect students' academic achievement in participating in learning, also can observe their ability to master the material, creativity, leadership, courage to take risks, and encouragement for entrepreneurship. They revealed this from the following lecturer's statement:

The success of mixed learning online and offline depends on the four competencies that lecturers must have. They are personality competence, where when an online learning lecturer's personality must be able to provide the best learning services and give a positive impression to students when participating in synchronous and asynchronous classes and face-to-face classes. Likewise, with pedagogic competence, the ability of lecturers to apply the method influences the students in understanding the material they get. They build social competence when lecturers can position themselves well in their student environment, have excellent speech, are polite in providing answers when answering student questions, and answer all questions asked by students without discrimination. Finally, professional compe-tence is a determinant of the competence of lecturers in using various learning media technologies that can improve material mastery, creativity, leadership in group work, dare to take risks, and increase entrepreneurial encoura-gement [Respondent L3].

The success of the PBOL method is even more significant if the lecturer has the four competencies above well. However, for learning to be easy and practical, lecturers need to use various other digital media, such as WhatsApp, Facebook, and YouTube, to facilitate real-time communication between lecturers and students. It also applied them to support the PBOL method if students have problems chatting through Microsoft Teams. Students revealed this statement during the following interview:

The PBOL method allows us to get alternative learning services through social media, such as WhatsApp, Facebook, and YouTube. It is necessary when we have problems asking and answering questions using Microsoft Teams or when we need faster feedback from lecturers. We can use this social media to support communication with lecturers so that the project-based task implementation process can run smoothly [Respondent S1].

Another challenge for lecturers in implementing the PBOL method in entrepreneurship learning for fifth-semester students is time allocation. The lecturer must devote more time to responding to all questions and student needs during discussions in the synchronous class and when learning to do assignments in the asynchronous. Lecturers must provide full-time to serve the learning process so that the time required is not too long. The lecturer should manage the time allocation for consultation or question and answer during asynchronous classes and when studying independently outside the classroom. Lecturers gave us six hours each day for the question-answer session outside the learning class from 9 am to 3 pm. So that learning takes place according to the schedule.

When we communicate with lecturers, we get feedback in real-time. Students can ask us as their lecturers directly every day from 9 am to 3 pm. We cannot provide real-time answers to questions outside these hours, but we usually send answers on the next day's schedule. We set this regulation to make students disciplined to carry out their tasks on time under the agreed learning consultation schedule [Respondent L4].

The lecturer's statement above shows that the determination of a learning consultation schedule for 6 h every day aimed at making students disciplined and able to take advantage of distance learning classes more effectively and efficiently. Internet signal factors also affect the effectiveness and efficiency of learning, especially during synchronous classes, where internet signals play an important role in smooth communication in the virtual classroom. The following is the lecturer's statement:

Synchronous virtual classroom learning can be successful if the lecturer is ready with stable internet materials and devices to support the implementation of online learning. The strength of this internet signal will determine the success of all students' connections during distance learning. The PBOL method used Microsoft Teams and social media supported by strong internet signals to make virtual discussion classes lively and fun. They are not only focused on understanding the material taught by the lecturer but also present their new ideas according to the knowledge they gained from self-study at home before entering the online virtual classroom [Respondent L2].

Students revealed during an interview with them through a zoom meeting also supported this statement.

The major problem we faced while studying entrepreneurship online was the instability of the internet, which could hinder smooth communication during discussions in virtual classes. We received an incomplete explanation from the lecturer when the internet signal was interrupted. Therefore, the stability of the internet and the availability of compatible technological devices will help our success in absorbing all the knowledge that lecturers teach us through digital media, which has a positive impact on improving academic achievement [Respondent S3].

The PBOL method used digital classroom devices of Microsoft Teams and social media in learning entrepreneurship material to make students easy, enthusiastic, and fun. When the internet signal during learning is strong and supported by high student engagement, it will provide a much better success rate for learning outcomes comprising concept mastery, creativity, leadership in group work, courage in taking risks/decisions, and increasing entrepreneurial encouragement to students.

#### 4.1.2 Quantitative finding

The measurement of the effectiveness of the PBOL method quantitatively to student academic achievement was by comparing the academic achievement of the PBOL class and conventional class after four months of doing exercises on digital business projects from August to December 2021 with the following results:

a. Student Academic Achievement Following the PBOL Method

The following result from the analysis of student academic achievement after four months of training on digital business projects using the PBOL method:

Table 1 shows the academic achievement of students attending entrepreneurship courses using the PBOL method. The indicator of concept mastery was 82.51 in the mean, with a standard deviation of 3.2729. The mean score for the creativity item was 83.24, with a standard deviation of 2.8338. The leadership item score was 84.47, with a standard deviation of 2.2556, all of which were in the excellent category. The risk-taking item has an average score of 79.73 and a standard deviation of 2.6886. Finally, the entrepreneurship encouragement item got an average score of 80.52, with a standard deviation of 3.3567. It is also in the excellent category.

b. Student Academic Achievement Following the Conventional Methods

**Table 1:** Academic achievement of PBOL class digital business project.

Indicators	Category			Mean	Standard Deviation
	Poor (0–50)	Good (51–79)	Excellent (80–100)		
Concept Mastery	3 (3.85%)	15 (19.23%)	60 (76.92%)	82.51	3.2729
Creativity	-	24 (30.77%)	52 (69.23%)	83.24	2.8338
Leadership	-	7 (8.97%)	69 (91.03%)	84.47	2.2556
Courage to take risks	2 (2.56%)	47 (60.26%)	29 (37.18%)	79.73	2.6886
Entrepreneurship encouragement	1 (1.28%)	30 (46.15%)	39 (52.56%)	80.52	3.3567
Average				82.09	

We got student academic achievements after participating in digital business project-based learning to use conventional methods:

Table 2 shows that the academic achievement of the entrepreneurship course for the concept mastery item is 74.78, with a standard deviation of 3.0206. For creativity, the mean score was 75.04, with a standard deviation of 3.2423. The average score of leadership items is 82.48, with a standard deviation of 3.3372. Both are in an excellent category. The items for the courage to take risks have an average score of 72.98 and a standard deviation of 3.3043. The last entrepreneurship encouragement item average score is 69.67 with a standard deviation of 2.9453 in the low category.

**Table 2:** Academic achievement of conventional class digital business project.

Indicators	Category			Mean	Standard Deviation
	Poor (0–50)	Good (51–79)	Excellent (80–100)		
Concept Mastery	17 (22.67%)	27 (36.00%)	31 (41.33%)	74.78	3.0206
Creativity	26 (36.47%)	36 (48.0%)	13 (17.33%)	75.04	3.2423
Leadership	6 (8.0%)	14 (18.67%)	55 (73.33%)	82.48	3.3372
Courage to take risks	38 (50.0%)	24 (31.6%)	14 (18.4%)	72.98	3.3043
Entrepreneurship encouragement	55 (73.33%)	17 (22.67%)	3 (4.00%)	69.67	2.9453
Average				74.99	

Of the five indicators for assessing student academic achievement in two treatment classes, both using the PBOL method and conventional, PBOL proved to be better than the conventional one under different accumulation values between the two groups studied.

**Figure 1:**
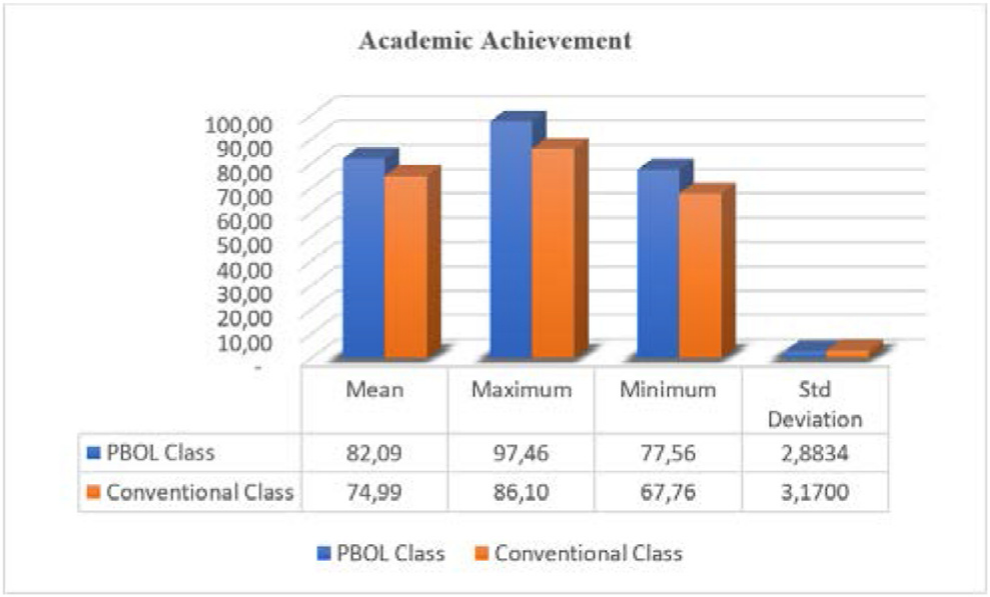
Academic achievement between PBOL class and conventional class.

Figure 1 indicates the mean score for the PBOL class was 82.09, with a standard deviation of 2.8834. The average value of the conventional one is 74.99, with a standard deviation of 3.1700.

### 4.2 Student engagement following the PBOL method

We can elaborate on the engagement of students in taking entrepreneurship courses using the PBOL method based on the findings of the following qualitative and quantitative data:

#### 4.2.1 Qualitative findings

The student engagement in learning using Microsoft Teams-based PBOL method and social media shows that learning that attracts attention can encourage student involvement to be more active in following it. This atmosphere improves the concentration of students after following all the teaching materials and exercises given by the lecturer in the synchronous and asynchronous class. Student engagement like this will positively affect the improvement of their learning outcomes.

Therefore, the attention and involvement of students is one factor for the success of a study. The higher a person's interest and engagement, the easier it is to concentrate and remember learning well. The following on the results of interviews with the lecturers in the field:

Using the PBOL method with Microsoft Teams digital class makes distance learning fun. This method facility convenient communication through chat rooms, easy download of materials, and ease of students reporting their assignments online through the assessment and grade feature in the Microsoft Teams digital class. In addition, it can accommodate various students to follow virtual face-to-face to involve actively during discussions in the synchronous class and follow-up activities in the asynchronous. Presentation of learning by lecturers and lively discussions during the process, and direct feedback provided by lecturers in real-time encourage students to be more actively involved in learning in class [Respondent L1].

One student who gave us a positive impression after following the PBOL method during the interview also revealed the same thing:

I enjoy taking entrepreneurship courses using this PBOL method. It makes it easier for me to understand the material. I also get various enrichments of material related to market opportunities, market management, and market strategy to determine a well-being market segment in building a digital business, including video tutorials shared by lecturers through digital classes. Interesting video tutorials supported by the flexibility of time that the lecturer gave us to practice every day according to a predetermined schedule made us feel relaxed and enjoy without the burden of doing project assignments given by the lecturer to do from home [Respondent S7].

The statement above revealed that the PBOL method used digital classes of Microsoft Teams with completed features for learning services, such as chatrooms, downloading materials, and uploading assignments and grades that provide direct feedback to students, coupled with social media facilities. The learning process is getting more interesting, especially the availability of various materials with various types of introductory media, ranging from text images to video tutorial media that students can access through Microsoft Teams and social media links provided by lecturers. In contrast to conventional learning, they do not get the ease of facilities and the attractiveness of the direct learning atmosphere at home.

Through an internet connection, lecturers and students can communicate anytime and anywhere related to the lecturer's efforts to increase students' understanding of the material they teach. Lecturers can answer student questions directly through Microsoft Teams or WhatsApp social media. In addition, through these two media, lecturers can monitor student activity in learning activities at home through the Facebook or YouTube link provided regarding strengthening the material. The more students interact and ask questions outside the classroom, it shows that they are more actively involved in independent learning from home, and this condition is not found in conventional classes, as expressed by the following lecturer:

Using the PBOL method based on Microsoft Teams digital classes supported by social media makes it easy for us to monitor student activities and involvement while participating. Synchronous and asynchronous online and face-to-face learning on campus can use Microsoft Teams and social media connected to digital classroom devices. It makes it more intense and encourages higher student learning involvement [Respondent L4].

Intensive strengthening and student involvement in online and offline learning highly depend on the readiness of the lecturers' dedication to their time to serving students while studying independently from home. This high dedication of time will support the effectiveness carried out by lecturers during online and offline learning. Meanwhile, student responses to the PBOL method based on digital classes using Microsoft Teams could have a significant influence on student engagement, as revealed from the results of interviews with several students as they:

This method is fun because, through the digital classes offered in this method using Microsoft Teams and social media, the nuances of two-sided learning direction are very pronounced when there is intensive communication in the synchronous class and the asynchronous class during distance learning [Respondent S6].

The expressions supporting this statement got from the lecturers during interviews with them.

I got a positive impression from students to access various learning materials and media that suit their needs, including downloading video tutorials to make it easier to understand digital business projects. They learn through a video tutorial and watch the video repeatedly until they master it [Respondent S4].

The statement above shows that the lecturer has successfully implemented this PBOL method well. The success of lecturers clearly cannot be separated from their competence in preparing better digital-based learning tools, especially in pedagogic and professional competencies. Besides social competence and personality, it is also a determining factor for students' interest, motivation and pleasure to be actively involved in learning what is taught by lecturers, both when participating in online learning and when taking part in face-to-face learning on campus.

Interestingly, the Microsoft Teams digital-based PBOL learning method and social media can support student concentration in learning. Students can choose various materials to refer to the type of digital business project they want to develop. Besides the material as tests, pictures, and videos, they can also get it through practice links, video links, and online material understanding competency test links. The availability of these innovative technology-based materials is of particular concern to students to be more interested in being actively involved in taking digital-based entrepreneurship courses using the PBOL method. They revealed this statement from the results of our interviews with the following students:

Entrepreneurship distance learning in a developmental line equipped with various digital learning media ranging from text and image-based materials to videos strongly supports increasing student engagement. Indeed, all of them encourage us as students to be interested in exploring the knowledge taught by the lecturers seriously and with a high concentration to answer the assigned tasks related to the material. Through this learning process, I no longer have to take notes like in a traditional class. However, I summarize the material provided by the lecturer and strengthen my understanding by reviewing the material repeatedly at home, applying, analyzing, and evaluating the material studied [Respondent S8].

This statement is parallel with the entrepreneurship lecturer utterance who was the respondent of this research, where during the interview, they said:

We used the PBOL method with Microsoft Teams and social media to make it easier to increase student interest and motivation in every online learning process in synchronous and asynchronous [Respondent L3].

The above statement revealed that the application of the PBOL method affects the students' engagement to be actively involved in every learning, neither behavioral, emotional, or cognitive involvement. As behavior changes, students appear more enthusiastic and disciplined by participating in online entrepreneurship courses through digital courses. They are fun about practicing independently at home every day according to the assignments given by the lecturer through distance learning.

Another emotional involvement effectiveness is that students feel comfortable and enjoy their learning, especially when working on assigned projects individually or in groups from their homes. Practicing alone will lead to a sense of self-confidence, responsibility, and top leadership in students. Lecturers evaluated the assessment of the student to get feedback related to the results of their project. If they still find errors, the lecturer will provide further guidance to revise their digital business assignment in the right creative economy sector.

The PBOL method is enjoyable during following distance learning. This method makes it easier the learning process of building digital business projects. I felt relaxed, free from the burden of not feeling pressured by the task [Respondent S1].

Likewise, the lecturer's statement in the following PBOL teaching method:

PBOL method using Microsoft Teams and social media proved to stimulate students' interest and motivation in being emotionally active in participating in this learning with feelings of pleasure and happiness following the entire learning process. Although it is time-consuming and tiring, they enjoy completing their respective projects and are always enthusiastic and actively involved in the learning process [Respondent L2].

The PBOL method also increases students' cognitive engagement after following distance learning. Students could understand the material presented easier by the lecturer in class, especially all the material presented by the lecturer used various types of digital media ranging from text images to video to trigger their cognitive engagement. They proved that almost all students actively ask questions if they do not understand the material. To overcome the limited face-to-face time on campus, the lecturer provides question-and-answer time service outside the classroom every day for 6 h, starting from 9 am to 3 pm. With the excellent service and real-time feedback, the cognitive engagement is getting higher during learning, although still a small part is low. They revealed this from the results of interviews with researchers with students:

Following the online study with the PBOL method improved my cognitive engagement because I was often involved in questions and answers with the lecturers when I did not understand some of the taught mater. Polite responses made me feel enjoyed and not afraid to ask the lecturers [Respondent S3].

The lecturer responded to this statement with a positive response:

The PBOL method with Microsoft Teams and social media opens up wider opportunities for all students to share discussions and ask questions about the material taught. It makes the discussion with colleagues or asking questions with the lecturer through the chat room communication media provided by the lecturer becomes easier [Respondent L1].

The PBOL method with Microsoft Teams and social media showed the optimal online learning process under-supported by digital classes that provide various student material needs in real-time, ranging from the material as text, images, or video tutorials. Everything makes it easier for students to learn independently. They feel happy, free from fear and anxiety because of the warmth of the lecturers, who are polite in answering their questions and responding directly to them in real-time. It makes them more enthusiastic about every online learning activity in synchronous and asynchronous classes. Learning experiences like this are not fully obtained by them in conventional classes.

#### 4.2.2 Quantitative finding of student engagement

Quantitatively, student involvement in following the PBOL method on student engagement shows the results:

Table 3 shows that the average student engagement is 4.04 in the high category, and the achievement percentage is 80.73%. It proved that students who take PBOL classes increased their involvement in learning. The highest average score for their curiosity behavior while attending lectures on item number one, which is 4.47 for the average Likert scale, and 89.5% of the achievement. They actively answer the lecturer's questions, express many new ideas, and enjoy discussions and debates during learning.

**Table 3:** Students' engagement in the PBOL method.

Not	Indicator	Likert scale	Percentage	Level
Behavioral Engagement				
1	I actively answer the lecturer's questions, express new ideas, and enjoy discussions and debates during online learning.	4.47	89.5	High
2	I actively observe, memorize, and follow the stages of building a business project through online learning.	4.39	87.9	High
3	I actively think and ask questions about things I don't understand in the virtual class and on social media.	4.17	83.4	High
4	I actively think associatively, comparing the difficulties I get while working on the project using PBOL.	4.34	86.8	High
5	I am actively improving my knowledge and skills related to working on business projects through the PBOL method.	3.08	61.6	Moderate
6	I diligently read to get information about business building tips and strategies independently through PBOL using Microsoft teams and social media.	4.44	82.9	High
7	I am actively asking about business feasibility calculations that I need to master through PBOL using Microsoft teams and social media.	4.29	86.8	High
8	I am more actively practicing doing business feasibility calculations in following the entrepreneurship subject through PBOL using Microsoft teams and social media.	4.20	83.9	High
	Average	4.17	82.85	High
9	I like entrepreneurship college through PBOL using Microsoft teams and social media because it can give me the skills to be a new entrepreneur.	3.07	61.3	Moderate
10	I like working on projects assigned by the lecturer under PBOL using Microsoft teams and social media.	4.04	80.8	High
11	I like to work together in groups doing project assignments assigned by the lecturer through PBOL using Microsoft teams and social media.	4.41	88.2	High
12	I like to learn to understand business-building strategies through video tutorials under PBOL using Microsoft teams and social media.	3.18	63.7	Moderate
13	I am happy to take entrepreneurship courses because they can improve my skills to become a prospective new entrepreneur, even learning through PBOL by using Microsoft teams and social media is fun.	4.43	88.7	High
14	I'm excited to join this project-based entrepreneurship online class.	4.26	85.3	High
	Average	3.90	78.00	Moderate
15	I am preparing to build a digital business through project-based lectures.	4.28	86.5	High
16	I concentrate and actively listen to the material in class through PBOL using Microsoft teams and social media.	3.04	60.8	Moderate
17	I am enthusiastic about participating in all entrepreneurship lecture activities, both online method and offline.	4.39	87.9	High
18	I am determined to be able to master the science of business and want to be a successful entrepreneur through the PBOL method.	4.25	85	High
19	I studied hard and tried to build a small business in the form of a college project through PBOL by using Microsoft teams and social media.	4.26	85.5	High
20	I attend every entrepreneurship lecture through the PBOL method using Microsoft teams and social media on time, both in virtual synchronous and asynchronous.	4.12	82.4	High
	Average	4.06	81.35	High
	Whole	4.04	80.73	High

The fourth indicator point: *I actively think associative, comparing the difficulties I get while working on the project using PBOL* with an average of 4.34 and a total achievement of 86.8%. It shows that behavioral engagement in entrepreneurship courses is in the high category. It contrasts with the fifth item: *I am actively improving my knowledge and skills related to working on business projects through the PBOL method*, with an average of 3.08 and achievement of 61.6%, the overall Likert scale behavior measurement is in the high category of 82.85%.

For the emotional attachment sub-variable, the highest score is item number 13: *I am happy to take entrepreneurship courses because they can improve my skills to become a prospective new entrepreneur. Learning through PBOL using Microsoft teams and social media is fun,* an average of 4.43 and a total achievement of 89.5%. Students were happily involved in entrepreneurship courses seen in item number nine: *I like entrepreneurship college through PBOL using Microsoft teams and social media because it can give me the skills to be a new entrepreneur*, an average of 3.07, and an achievement of 61.3%. The overall emotional involvement of students got an average of 3.90, and the achievement of 78.0% was at a moderate level.

The seventeenth sub-variable item of cognitive engagement shows the highest value of 4.39 and 87.9% for the statement: *I am enthusiastic about participating in all entrepreneurship lecture activities in online and offline methods*. It shows high students' cognitive engagement doing business entrepreneurship exercises assigned by lecturers. The lowest Likert scale shows an average of 3.04 and an achievement of 60.8% for question number 16: *I concentrate and actively listen to the material in class*. This item belongs to the medium category. Overall, the cognitive engagement indicator shows an average of 4.04, with a notable achievement of 80.73%.

Based on the three subcategories of student engagement observed above, the accumulated results of student engagement are:

Table 4 reveals engagement in entrepreneurship education classes with the PBOL method shows lower behavior, at 19 people (25.0%) and high at 57 (75.0%). For emotional behavior was ten people (13.2%), the medium was 17 people (22.4%) and the high, 49 people (64.5%), and finally for cognitive engagement group showed low cognitive behavior, two people (2,6%) were moderate, 19 people (25.0%) and 55 people (72.4%) were high. The average was 4,080 out of five Likert scale points in the high category.

**Table 4:** Student engagement following the PBOL method.

Student Engagement	Category			Means	Level
	Low (1.00–2.33)	Moderate (2.34–3.66)	High (3.67–5.0)		
Behavior	-	19 (25.0%)	57 (75.0%)	4.34	High
Emotional	10 (13.2%)	17 (22.4%)	49 (64.5%)	3.56	Moderate
Cognitive	2 (2.6%)	19 (25.0%)	55 (72.4%)	4.32	High
Average				4.08	High

Table 5 reveals student involvement in conventional classes where behavioral involvement is low: 15 people (19.7%), 60 people (78.9%) were moderate, and high 3 (3.9%). For emotional behavior in the low category, 13 people (17.2%), 60 people (78.9%) were moderate, and three people (3.9%) were high. Finally, for the low cognitive involvement group, there were 26 people (34.2%), moderate 48 people (63.2%), and two people with high (2.26%), and overall, the average got was 2,940 out of 5 points on Likert scale, which is in the medium category.

**Table 5:** Students' engagement following the conventional method.

Student Engagement	Category			Means	Level
	Low (1.00–2.33)	Moderate (2.34–3.66)	High (3.67–5.0)		
Behavior	15 (19.7%)	58 (76.4%)	3 (3.9%)	2.95	Moderate
Emotional	13 (17.2%)	60 (78.9%)	3 (3.9%)	3.00	Moderate
Cognitive	26 (34.2%)	48 (63.2%)	2 (2.6%)	2.87	Moderate
Average				2,94	Moderate

### 4.3 The influence of the application of the PBOL method and student involvement on students' academic achievement

We analyzed the PBOL method and student engagement effect on their academic achievement using Two Way ANOVA analysis (see Table 6).

**Table 6:** Effect of the implementing PBOL method and student engagement.

Dependent Variable:Academic Achievement					
Source	Type III Sum of Squares	df	Mean Square	F	Sig.
Corrected Model	4629,706a	5	925,941	7.099	.000
Intercept	899104,581	1	899104,581	6.894E3	.000
Method	1092.817	1	1092.817	8,379	.004
Engagement	1006.183	2	503.092	3.857	.023
Method ∗ Engagement	2471,360	2	1235,680	9,474	.000
Error	19172,765	147	130,427		
Total	924483,000	153			
Corrected Total	23802.471	152			
a. R Squared = .195 (Adjusted R Squared = .167)					

Table 6 indicates the two-way ANOVA analysis shows that the PBOL method is more effective than the conventional method. These findings reveal that the effect of the learning method on student academic achievement based on learning method was a significance value of 0.004 and F = 8.379. The student engagement variable on academic achievement shows a significance value of 0.023 and an F value of 3.857, and the interaction between learning methods and engagement was 0.000, F-value of 9.477.

Table 7 Indicates the post-ANOVA analysis above shows an interaction between the method and student engagement in entrepreneurial academic achievement.

1. Post-ANOVA analysis did not find any difference in student academic achievement between high and moderate student engagement with a significance value of 0.939.
2. Post-ANOVA analysis did not find any difference in student academic achievement between moderate and low student engagement, with a significance value of 0.061.
3. Post-ANOVA analysis found differences in student academic achievement between low and high student engagement, with a significance value of 0.026.

**Table 7:** Post-ANOVA follow-up test using scheffe.

Academic Achievements Tukey HSD						
(I) Student Engagement	(J) Student Engagement	Mean Difference (IJ)	Std. Error	Sig.	95% Confidence Interval	
					Lower Bound	Upper Bound
High	Moderate	.7647	2.26159	.939	-4.5900	6.1195
	Low	5.9412∗	2.26159	.026	.5864	11.2959
Moderate	High	-.7647	2.26159	.939	-6.1195	4.5900
	Low	5.1765	2.26159	.061	-.1783	10.5312
Low	High	-5.9412∗	2.26159	.026	-11.2959	-.5864
	Moderate	-5.1765	2.26159	.061	-10.5312	.1783

## 5 Discussion

The study results revealed that the PBOL method in class influenced student engagement during lectures using Microsoft Teams and social media, such as WhatsApp, Facebook, and YouTube. In addition, the results show that there is an interaction between the method and student involvement with academic achievement. This finding strongly supports the results of qualitative analysis where learning activities and student responses during entrepreneurship lectures with the PBOL method show a positive response. They feel happy, enjoy, and do not feel burdened when attending entrepreneurship lectures. This condition has an impact on increasing the behavioral, emotional and cogntiive engagement of students. The successful learning of the PBOL method is very effective in providing real-time feedback directly to students when they ask questions. Another effectiveness, this method makes it easier for students and lecturers to communicate remotely and makes it easier for lecturers to provide complete explanations with links to the material needed by students.

This research similarity with Hogue and Kapralos's (2011) revealed that the PBL method has improved students' skills and broadened their theoretical and practical knowledge. With the technology integration learning approach into the entrepreneurship curriculum, researchers have seen higher retention rates, increased motivation, and quality of student work experience significant improvements. All involved respondents revealed that the PBOL method succeeds increased their involvement in entrepreneurship courses. It also found that almost all students were interested in taking classes using this PBOL method. It affects increasing the active engagement of students in each of their activities. The lecturers are happy with the activeness of their students in completing all the entrepreneurship project assignments. Remarkable interestingly, through the PBOL method, all students can directly work on digital-based business projects they design as their last task. Safaruddin et al. (2020) revealed that project-based learning with electronic media could improve learning motivation and the scientific process. This result reported that this method could increase student learning motivation in the excellent category 78.05.

All students complete the entrepreneurship course final project under the guidance of the lecturer. It encourages them to revive their entrepreneurial spirit and want to become successful entrepreneurs who can change their destiny, no longer hoping to become staff, but want to become successful entrepreneurs who employ many employees. This finding supported the research of Reno et al. (2020) said that the use of media zoom, Microsoft Teams, Microsoft OneDrive, and social media are the most popular applications widely in the usage of various businesses and are compatible with the PBL method.

Lambert and Rennie (2021) reported that distance learning for entrepreneurship courses was quite effective in using social media to increase student engagement and academic achievement. Attendance issues increase between group work through Microsoft Teams media. The student engagement gain was 3.94, and the average was 78.8%. It means extraordinary achievements that can encourage the entrepreneurial spirit of students appeared. It shows that the effectiveness of the PBOL method of 97.6% can increase student engagement between 60.8-89.5% in the excellent category (see Table 3). It supported Okudan and Rzasa (2006) in a project-based approach to entrepreneurial leadership education. They reported that they could encourage students' academic achievement to complete entrepreneurial projects and generate financial returns. One team made a profit of around $700, a remarkable achievement considering the relatively short time learning.

The application of the PBOL method is one of the learning methods that require students to discuss in their group links using social media. From the results of observations of student learning activities, all aspects or indicators assessed show the achievement of extraordinary learning success targets.

Erwantiningsih et al. (2021) revealed that Project-Based Learning on the independence of students' interest in entrepreneurship has a significant influence of 38.2% contribution. Research from Paľová et al. (2020) revealed that implementing project-based learning and virtual laboratories supports entrepreneurship education in the two study programs offered by the economics faculty. Through project-based learning, participants in this course gain the opportunity to apply their knowledge, skills, and experience to solve practical problems. They also have direct experience and manage small virtual businesses within the virtual laboratory of entrepreneurship education. The findings show that project-based learning and virtual laboratories support the achievement of electronic media-based entrepreneurship education for the students' entrepreneurial skills development.

Dragoumanos et al. (2017) revealed that the Project-Based Learning method on student entrepreneurial interest in the Applied Informatics in Business and Economics program showed that 80 percent were active participants. They feel confident in their knowledge and can start their own business. An interesting finding is that 40% of students have already built a team to develop their product, and they all believe can their entrepreneurship skills develop during their studies. The written test showed that their hard skills had also improved. Okudan and Rzasa (2006) also revealed that the PBL method could enhance leadership learning in entrepreneurship through projects to build and sell products for profit. It proved that the project-based learning process in entrepreneurship courses could provide direct experience in building a business among students. Ariyani and Zuhaery (2021) revealed that leadership-based entrepreneurial learning uses two fundamental principles in creating a learning environment in the school. First, the principal regulates organizational growth through optimization, communication, motivation, monitoring, control, example, and empowerment. Second, the principal made several innovations through several steps: vision development, staff development, and restructuring. This kind of leadership-based entrepreneurial learning can create a comfortable and fun learning environment in the learning process to improve and make some progress in academic and non-academic achievements.

Botha (2010) revealed that implementing project-based learning indicated greater student satisfaction with the method used. In addition, the assessment of student interest shows their positive perception of acquiring entrepreneurial skills during the project. Panfilova et al. (2019) also revealed that the application of the PBL showed that student participation in real projects increased their professional skills by 6%–10%. Also, raise their high entrepreneurial interest. The PBL method for lecturers has a positive reaction of 67% to students' interest in participating in real project-based training. The results indicate that real project-based learning in higher education is quite effective as a digital-based entrepreneurial learning approach.

A comparison of the final results between the PBOL and Conventional methods in digital-based entrepreneurship lectures reveals that learning with the PBOL method is excellent, illustrating that an average score of 82.09 participants takes the course. This figure is higher than the conventional class of 74.99 (see Figure 1). In addition, they feel they can lead the business (91.03%) were confident without fear of risk (79.73%), with a mastery of theory (76.92%), and high creativity (69.23%). An interesting finding was that 52.56% of students experienced a high level of interest and motivation to develop their own business than the conventional class achievement of 18.4% (see Tables 1 and 2). They all believe that entrepreneurship skills through learning lectures using the PBOL method can improve their academic achievements better than conventional ones.

Consistent with Guo et al. (2020) found four categories out of seven subcategories of student learning outcomes in PBL: Cognitive (knowledge, cognitive strategies), affective (perception of the benefit of PBL, perception of the experience of PBL), Behavioral outcome (Skills, Engagement) and artifact performance (physical object, documents, and multimedia) can improve student entrepreneurship learning outcomes in higher education.

This study differs from the findings of Burnette et al. (2020) revealed that PBL increases students' interest in growth mindset interventions in entrepreneurship courses and self-efficacy and career development (i.e., academic interest, career interest, task persistence, and academic performance). However, the intervention fails to directly or indirectly impact class assignment performance.

Leal Filho et al. (2016) revealed that project-based learning could provide experiences in real-world learning scenarios that allow for better competence development, not only to be appreciated in the workplace but will be vital for students' self-development. PBL will improve sustainability literacy in student learning on campus. Therefore, the digital-based PBL methods strengthen institutional learning and integrative learning approach to entrepreneurship education in the future.

Likewise, the comparison of active student involvement during lectures using the PBOL method is better at 4.08 on a five-point Likert scale in student engagement than the conventional class of 2.94. The student engagement during entrepreneurship lectures shows that the behavior in all learning and entrepreneurship assignments to the PBOL is higher (4.34) than in conventional class (2.95). Remarkably, the cognitive abilities in the PBOL class are much better in their academic achievement (4.32) than in conventional ones (2.87), although emotional involvement is not much different (see Tables 4 and 5). It shows that behavioral engagement affects students' academic achievement during entrepreneurship courses.

This study differs from Simpson's (2013) revealed that students enrolled in distance education courses tend to have lower course completion rates than those who attend face-to-face classes. Meanwhile, Yates et al. (2020) reported on the contrary that online learning can increase student engagement and high learning completion rates. The students could complete their distance studies well is supported by the competence of teachers in overcoming obstacles encountered during online learning. In addition, the ability of lecturers to create a learning atmosphere well and the willingness of the lecturers to provide full time to serve the entire distance learning process and always provide real-time feedback on all progress in understanding and mastery of concepts, creativity, leadership in group assignments, courage to take risks and Entrepreneurship encouragement which are expected to increase their desire to become new entrepreneurs.

Sousa et al. (2019) reported that the use of digital methodologies in education, as in all the studies made in the last three years reported that technology can improve student learning processes with innovations using mobile technology, tablets, and smartphone applications. It leads students to work collaboratively, communicate efficiently and autonomously in the learning process and become independent to create their own businesses or become entrepreneurs in professional life. A student with sustainable entrepreneurial ideas, the use of e-education methods enable their success by increasing engagement in learning network, cooperative learning, and direct student involvement in the business world.

Meanwhile, there is an interaction between the method and student engagement in student entrepreneurship learning achievement. The post-ANOVA demonstrated an interaction between low engagement, moderate and high levels in entrepreneurial academic achievement, where no difference in student academic achievement between high and low student engagement with a significance value of 0.939 and moderate and low level of student engagement, with a significance value of 0.061. Meanwhile, the prediction results showed any interaction between low student engagement and high level to academic achievement, with a significance value of 0.026 (see Table 7).

Siddiq et al. (2020) and Santosa (2015) reported a positive interaction between the online learning method and student engagement in learning outcomes seems vividly. The behavior of using online learning is a manifestation of student engagement. Online learning by using these media shows how technology affects student engagement in online teaching. This interaction showed that the PBOL learning method and student engagement affect student academic achievement. This interaction still affects the intensity of students' learning engagement itself. These findings conclude that lower learning engagement cannot improve students' academic achievement in entrepreneurship courses using the PBOL method.

Qualitative findings reported success of the PBOL method is highly dependent on the ability of lecturers to create fun and communicative entrepreneurship lectures equipped with adequate digital facilities. Another factor was the lecturers' ability to overcome online lecture problems, especially ensuring stable internet signal during learning and digital devices reliability, and the willingness of the lecturers to provide full time to serve the entire distance learning process. They also should provide real-time feedback on all student learning progress. Tarman et al. (2019) revealed that the experienced barriers to digital learning are lack of technological skills, limited internet access, and administrative and technical use of the program.

Secundo et al. (2021) reported that the implementation of technology-based entrepreneurship education in universities during the COVID-19 pandemic needs to highlight the strengths of the designed programs, particularly the limitations of digital technology in education, which represent areas for future improvement. They concluded that digitally supported entrepreneurship education provides new insights into redesigning traditional university programs to implement digital-based learning during the COVID-19 emergency.

## 6 Conclusion

Our findings conclude that the PBOL method uses Microsoft Teams and social media effectively could improve student engagement and academic achievement. It could create an enjoyable learning atmosphere because learning services from lecturers are friendly, polite, and quick to provide answers when students ask questions through social media. It makes more communicative entrepreneurship lectures equipped with adequate digital facilities reliability including the lecturers' ability to overcome online lecture problems, ensure stable internet signal during learning, and the willingness of the lecturers to provide full time to serve the entire distance learning process well. Quantitatively, these show an effect of the PBOL method and student involvement on academic achievement, and interaction between the method with high and low student involvement in academic achievement occurred. The effectiveness of learning the PBOL method and student engagement in improving entrepreneurial skills is necessary for lecturers' competence in creating quality virtual "experience" classrooms supported by the availability of entrepreneurship learning equipment facilities with complete PBOL methods and supported by a robust internet signal, and learning material mastery by lecturers well.

## 7 Recommendation

The conceptual challenge for future academics is to design online project-based educational curricula in such a way as to enable knowledge to be applied in pursuing digital-based sustainability projects that are currently developing. PBOL should apply carefully in the context of the university to bring benefits to the sustainability of entrepreneurship education in the future. We suggest that entrepreneurship lecturers in universities apply the PBOL method developed to increase student engagement and academic achievement by using the digital platform social media and Microsoft Teams. This method can be an alternative for lecturers in carrying out the entrepreneurship learning process in the new-normal period, where there are still restrictions on face-to-face classes.

## 8 Limitations

This study has several limitations: the PBOL method only provides online learning solutions for entrepreneurship courses according to their needs. These findings are not representative of all assessment subjects on a large scale. In addition, the limited time allocation in project completion and further monitoring of the development of student entrepreneurial interests to build a business independently require a long-term observation period to determine the success of academic achievement of students.

## Declarations

### Author contribution statement

Zelhendri Zen, conceived and designed the experiments; Performed the experiments; Wrote the paper.

Reflianto Reflianto, Analyzed and interpreted the data; Wrote the paper.

Syamsuar Syamsuar, Performed the experiments; Contributed reagents, materials, analysis tools or data.

Farida Ariani, Analyzed and interpreted the data; Wrote the paper.

### Funding statement

This research did not receive any specific grant from funding agencies in the public, commercial, or not-for-profit sectors.

### Data availability statement

Data included in article/supp. material/referenced in article.

### Declaration of interest's statement

The authors declare no conflict of interest.

### Additional information

No additional information is available for this paper.

## Appendix A Supplementary data

Supplementary Material- Interview Protocol and Questioner Supplementary Material- Interview Protocol and Questioner

## References

[bib1] Aadland T., Aaboen L. (2020). An entrepreneurship education taxonomy based on authenticity. Eur. J. Eng. Educ..

[bib2] Arias E., Barba-Sánchez V., Carrión C., Casado R. (2018). Enhancing entrepreneurship education in a master’s degree in computer engineering: a project-based learning approach. Adm. Sci..

[bib3] Ariyani D. Suyatno, Zuhaery M. (2021). Principal’s innovation and entrepreneurial leadership to establish a positive learning environment. Eur. J. Educ. Res..

[bib4] Attard C., Holmes K. (2020). It gives you that sense of hope”: an exploration of technology use to mediate student engagement with mathematics. Heliyon.

[bib5] Belwal Rakesh, Belwal Shweta, Sufian Azlinor Binti, Al Badi, Amal (2020). Project-based Learning (PBL): Outcomes of students’ engagement in an external consultancy project in Oman. Educ. Train..

[bib6] Boss S., Krauss J. (2014).

[bib7] Botha M. (2010). A project-based learning approach as a method of teaching entrepreneurship to a large group of undergraduate students in South Africa. Educ. Change.

[bib8] Britten N. (1995). Qualitative research: qualitative interviews in medical research. Br. Med. J..

[bib9] Bryer T.A., Zavattaro S.M. (2011). Social media and public administration: theoretical dimensions and introduction to the symposium. Adm. Theor. Prax..

[bib10] Burnette J.L., Pollack J.M., Forsyth R.B., Hoyt C.L., Babij A.D., Thomas F.N., Coy A.E. (2020). A growth mindset intervention: enhancing students’ entrepreneurial self-efficacy and career development. Enterpren. Theor. Pract..

[bib11] Carnawi C., Sudarmin S., Wijayati N. (2017). Application of project based learning (PBL) model for materials of salt hydrolysis to encourage students’ entrepreneurship behaviour. Int. J. Active Learn..

[bib12] Cooper J.M. (2010).

[bib13] Creswell J.W., Clark V.L.P. (2017).

[bib14] Creswell W J. (2014).

[bib15] Crosling G., Nair M., Vaithilingam S. (2015). A creative learning ecosystem, quality of education and innovative capacity: a perspective from higher education. Stud. High Educ..

[bib16] Denzin N. (2020). https://www.unaids.org/sites/default/files/sub_landing/files/10_4-Intro-to-triangulation-MEF.pdf.

[bib17] Dilley P. (2004). Interviews and the philosophy of qualitative research. J. High Educ..

[bib18] Dorn E., Hancock B., Sarakatsannis J., Viruleg E. (2020).

[bib19] Dragoumanos S., Kakarountas A., Fourou T. (2017). 2017 IEEE Global Engineering Education Conference (EDUCON), 351–358.

[bib20] Erwantiningsih E., Wahyuni H., Immadudin W. (2021). Developing entrepreneurial interest and student independence through project-based entrepreneurship learning. Pedagogia: J. Pendidikan.

[bib21] Guo P., Saab N., Post L.S., Admiraal W. (2020). A review of project-based learning in higher education: student outcomes and measures. Int. J. Educ. Res..

[bib22] Han Z., Wang Q., Yan X. (2019). How responsible leadership motivates employees to engage in organizational citizenship behavior for the environment: a double-mediation model. Sustainability.

[bib23] Hart S.R., Stewart K., Jimerson S.R. (2011). Contemporary School Psychology: Formerly" The California School Psychologist.

[bib24] Herdjiono I., Puspa Y.H., Maulany G., Aldy E. (2017). The factors affecting entrepreneurship intention. Int. J. Entrepren. Knowl..

[bib25] Hogue A., Kapralos B. (2011). The role of project-based learning in IT: a case study in a game development and entrepreneurship program. Interact. Technol. Smart Educ..

[bib26] Kahu E.R. (2013).

[bib27] Lambert C.G., Rennie A.E.W. (2021). Experiences from COVID-19 and emergency remote teaching for entrepreneurship education in engineering programmes. Educ. Sci..

[bib28] Leal Filho W., Shiel C., Paço A. (2016). Implementing and operationalising integrative approaches to sustainability in higher education: the role of project-oriented learning. J. Clean. Prod..

[bib29] Lokey-Vega A., Bondeson K., Resta P., Smith S. (2017). Proceedings of Society for Information Technology & Teacher Education International Conference.

[bib30] Majid M.A.A., Othman M., Mohamad S.F., Lim S.A.H., Yusof A. (2017). Piloting for interviews in qualitative research: operationalization and lessons learnt. Int. J. Acad. Res. Bus. Soc. Sci..

[bib31] Martín P., Potočnik K., Fras A.B. (2017). Determinants of students’ innovation in higher education. Stud. High Educ..

[bib32] Okudan G.E., Rzasa S.E. (2006). A project-based approach to entrepreneurial leadership education. Technovation.

[bib33] Paľová D., Vejačka M., Kakalejčík L. (2020). Project-based learning as a tool of enhancing of entrepreneurial attitude of students. Adv. Sci. Technol. Eng. Syst. J..

[bib34] Pan G., Seow P.-S., Koh G. (2019). Examining learning transformation in project-based learning process. J. Int. Educ. Bus..

[bib35] Panfilova E.E., Demkina O.V., Galichkina M.A., Istomina A.I., Latysheva V.V., Teymurova V.E. (2019). Learning models based on a real project in entrepreneurial education. J. Enterpren. Educ..

[bib36] Phothongsunan S. (2020). Student and teacher engagement in learning and assessment with portfolios. Cypr. J. Educ. Sci..

[bib37] Rasulzada F. (2017). Research Handbook of Innovation and Creativity for Marketing Management.

[bib38] Raza S.A., Qazi W., Umer B. (2019). Examining the impact of case-based learning on student engagement, learning motivation and learning performance among university students. J. Appl. Res. High Educ..

[bib39] Reflianto, Setyosari P., Kuswandi D., Widiati U. (2021). Reading comprehension skills: the effect of online flipped classroom learning and student engagement during the COVID-19 pandemic. Eur. J. Educ. Res..

[bib40] Reno L.S.E., Harmon L., Hutto L., McManus K., Hochradel R., Stovall T. (2020). Proceedings of A Virtual Experience, Marketing Management Association Fall Educators’ Conference.

[bib41] Rose J., Johnson C.W. (2020). Contextualizing reliability and validity in qualitative research: toward more rigorous and trustworthy qualitative social science in leisure research. J. Leisure Res..

[bib42] Safaruddin S., Ibrahim N., Juhaeni J., Harmilawati H., Qadrianti L. (2020). The effect of project-based learning assisted by electronic media on learning motivation and science process skills. J. Innov. Educ. Cult. Res..

[bib43] Samuel A.B., Rahman M.M. (2018). Innovative teaching methods and entrepreneurship education: a review of literature. J. Res. Bus. Econ. Manag..

[bib44] Santosa P.I. (2015). Student engagement with online tutorial: a perspective on flow theory. Int. J. Emerg. Technol. Learn. (IJET).

[bib45] Sayan H. (2016). Affecting higher students learning activity by using WhatsApp. Eur. J. Res. Reflect. Educ. Sci..

[bib46] Secundo G., Gioconda M., Del Vecchio P., Gianluca E., Margherita A., Valentina N. (2021). Threat or opportunity? A case study of digital-enabled redesign of entrepreneurship education in the COVID-19 emergency. Technol. Forecast. Soc. Change.

[bib58] Setyosari P., Kuswandi D., Widiati U. (2022). English teachers’ competency in flipped learning: question level and questioning strategy in reading comprehension. Int. J. Instruct..

[bib47] Shin N., Bowers J., Krajcik J., Damelin D. (2021). Promoting computational thinking through project-based learning. Disc. Interdisc. Sci. Educ. Res..

[bib48] Siddiq F., Gochyyev P., Valls O. (2020). The role of engagement and academic behavioral skills on young students’ academic performance—a validation across four countries. Stud. Educ. Eval..

[bib49] Simpson O. (2013). Student retention in distance education: are we failing our students?. Open Learn.: J. Open Dist. E Learn..

[bib50] Skinner E.A., Pitzer J.R. (2018). Handbook of Research on Student Engagement.

[bib51] Sousa M.J., Carmo M., Gonçalves A.C., Cruz R., Martins J.M. (2019). Creating knowledge and entrepreneurial capacity for HE students with digital education methodologies: differences in the perceptions of students and entrepreneurs. J. Bus. Res..

[bib52] Starko A.J. (2017).

[bib53] Suartama I., Setyosari P., Sulthoni S., Ulfa S. (2020). Development of ubiquitous learning environment based on moodle learning management system. Int. J. Interact. Mobile Technol. (iJIM).

[bib54] Tarman B., Kilinc E., Aydin H. (2019). Barriers to the effective use of technology integration in social studies education. Contemp. Issues Technol. Teach. Educ..

[bib55] Verdugo G.B., Villarroel A.V. (2021). Measuring the association between students’ exposure to social media and their valuation of sustainability in entrepreneurship. Heliyon.

[bib56] Virtanen M.A., Haavisto E., Liikanen E., Kääriäinen M. (2018). Ubiquitous learning environments in higher education: a scoping literature review. Educ. Inf. Technol..

[bib57] Yates A., Brindley-Richards W., Thistoll T. (2020). Student engagement in distance-based vocational education. J. Open Flex. Dist. Learn..

