# Supplementary material for: Academic achievement: the effect of project-based online learning method and student engagement
Source: Heliyon. 2022 Nov 12;8(11):e11509. doi: 10.1016/j.heliyon.2022.e11509 (PMC9674908; doi:10.1016/j.heliyon.2022.e11509)
Supplement: Supplementary Material- Interview Protocol and Questioner [file mmc1.docx]

**Supplementary Material**

**Lecturer Participant Informed Consent Form**

I, ______________________________, agree to participate in in a research study The Effect of Project-Based Online Learning Methods (PBOL) and Student Engagement on Entrepreneurship Learning Outcomes. The purpose of this study is to obtain the perspectives of lecturers and students about the effectiveness of the PBOL model and their level of learning engagement when attending entrepreneurship classes and to analyze whether there is an effect of applying the Project Based Online Learning method and student engagement on increasing student entrepreneurship learning outcomes at the Faculty of Education, State University of Padang.

As a lecturer who carries out entrepreneurship lectures in this research, I understand that my participation as a lecturer in this research is purposeful and voluntary. I am willing to provide a perspective on my experience of implementing PBOL in the entrepreneurship course during four months of research in an interview with the principal investigator. I understand that 4 lecturers will be selected to participate in this research and a total of 153 students will be involved.

I recognised and understand that the research assistant, Dr. Reflianto, M.Pd has received training on research of human subject, my responses will remain confidential, and that my name will never be associated with any results from this research. I will be labeled with a number, identifiable only by the researcher and participant. I understand that the data collection process will include the use of audio recording devices and all recordings will be transcribed for analysis. I understand that my identity will not be disclosed or even associated with the research findings.

I agree that I am free to withdraw my consent to participate in this study. If I choose to do so, I agree that I will notify the researchers listed below, in writing. If I decide to withdraw from research participation, there will be no effect on my relationship with the researcher.

I understand that in return for my participation in all interviews, and filling out the questionnaire, I will be provided a nutritional lunch with the principal investigator at the culmination of the study.

If I have any questions or concerns that may arise as a result of my participation in teaching, I understand that I should contact the principal investigator, Drs. Zelhendri Zen, M.Pd at +6281363840916 or zelhendrizen@fip.unp.ac.id

Other contacts include research assistant, Dr. Reflianto, M.Pd at +6281363026186 or refliantomuslim@gmail.com and Drs. Syamsuar, MS. AIFO. PhD at 6281286387757 or syamsuar.unp@fik.unp.ac.id

My signature below affirms that I am at least 18 years of age, that I have received a copy of

consent form, and that I agree to participate in this study.

_____________________________________ Signature of Lecturer Participant

_____________________________________ Date

_____________________________________ Signature of Researcher

_____________________________________ Date

**Student Participant Informed Consent Form**

I, ______________________________, agree to participate in in a research study The Effect of Project-Based Online Learning Methods (PBOL) and Student Engagement on Entrepreneurship Learning Outcomes. The purpose of this study is to obtain the perspectives of lecturers and students about the effectiveness of the PBOL model and their level of learning engagement when attending entrepreneurship classes and to analyze whether there is an effect of applying the Project Based Online Learning method and student engagement on increasing student entrepreneurship learning outcomes at the Faculty of Education, State University of Padang.

As a student participating in the entrepreneurship course in this research, I understand that my participation in this research is purposeful and voluntary. I am willing to provide perspective on my experience following the PBOL learning method in entrepreneurship class both during interviews and when asked to fill out a questionnaire. I understand that 4 lecturers will be selected to participate in this research and a total of 153 students will be involved.

I recognised and understand that the research assistant, Dr. Reflianto, M.Pd has received training on research of human subject, my responses will remain confidential, and that my name will never be associated with any results from this research. I will be labeled with a number, identifiable only by the researcher and participant. I understand that the data collection process will include the use of audio recording devices and all recordings will be transcribed for analysis. I understand that my identity will not be disclosed or even associated with the research findings.

I agree that I am free to withdraw my consent to participate in this study. If I choose to do so, I agree that I will notify the researchers listed below, in writing. If I decide to withdraw from research participation, there will be no effect on my relationship with the researcher.

I understand that in return for my participation in all interviews, and filling out the questionnaire, I will be provided a nutritional lunch with the principal investigator at the culmination of the study.

If I have any questions or concerns that may arise as a result of my participation in teaching, I understand that I should contact the principal investigator, Drs. Zelhendri Zen, M.Pd at +6281363840916 or zelhendrizen@fip.unp.ac.id

Other contacts include research assistant, Dr. Reflianto, M.Pd at +6281363026186 or refliantomuslim@gmail.com and Drs. Syamsuar, MS. AIFO. PhD at 6281286387757 or syamsuar.unp@fik.unp.ac.id

My signature below affirms that I am at least 18 years of age, that I have received a copy of

consent form, and that I agree to participate in this study.

_____________________________________ Signature of Student Participant

_____________________________________ Date

_____________________________________ Signature of Researcher

_____________________________________ Date

**Appendix 1**

**Lecturer Interview Protocol**

**Project:**          The Effect of Project-Based Online Learning Methods (PBOL) and Student Engagement on Entrepreneurship Learning Outcomes

Interview Time :

Date :

Place : Padang State University

Interviewer : Drs. Zelhendri Zen, M.Pd. PhD

Interviewee :

Resource Person Position : Entrepreneurship Lecturer

Thank you for taking the time to talk to me about your perception of online lectures in entrepreneurship classes at the Padang State University Faculty of Education students.

You were selected to participate because you are currently a lecturer in entrepreneurship courses at the Faculty of Education, State University of Padang. To improve the results and quality of student learning in entrepreneurship courses, this research study is designed to provide a platform so that your voice, opinion, and input on the impact of applying the PBOL method and student engagement on student learning outcomes that you experience in your entrepreneurship class can be heard. revealed.

Please note that there are no right or wrong answers to these questions. My role is simply to record information and share information that offers a glimpse of your experience in the entrepreneurship class.

Please note that all of your responses will remain confidential, and identifying information will be removed from the transcript. You may withdraw from this interview at any time without penalty. Your responses to these interview questions will form part of my research into the adoption of the PBOL method and student engagement in learning outcomes in entrepreneurship classes.

Our interview today should take no more than 30 minutes. As you'll notice, I rigorously recorded audio of our session to help me transcribe and analyze your feedback. After the interview is transcribed, I will provide a transcript, or a detailed document that includes your responses, so you can verify its accuracy.

[Confirm that I have received the student consent form ahead of the interview.]

[Turn on the digital recorder and test it.]

_____________ (lecturer), do you have any questions before we start?

Initial Lecturer Interview Questions:

Introduction: Let’s begin with some background information on what it means to be engaged. Being “engaged” considers three forms: behavioral/social engagement, emotional engagement, and cognitive engagement. Behavioral/social engagement includes how you demonstrate “the behaviors expected in a classroom—such as listening, doing assignments, following directions, participating, and so on” (Cooper, 2014, p. 365). Additionally, emotional engagement includes your sense of positivity for a class, your feelings of being satisfied, comfortable and interested, and your desire to find success in entrepreneurship class (Blumenfeld et al., 2006; J. A. Fredricks et al., 2004). And finally, cognitive engagement includes the amount of mental energy you exert, the ways that you are thinking about entrepreneurship content, and the ways you investigate new information and work with mental challenges (Cooper, 2014).

By understanding student engagement, explain what it means when a student is "engaged" in their learning in an entrepreneurship class by using Project Based Online Learning Method. Please give your response to our questions :

1. How did the lecturers conduct entrepreneurship lectures using the PBOL method during the COVID-19 pandemic?
2. Describe your experience with students’ behavior, feelings, and impressions following the entrepreneurship class by using the PBOL method supported by Microsoft Team and social media?
3. As a lecturer when you are teaching actively the entrepreneurship subject both in synchronous and asynchronous classes using the PBOL method, What is your perspective about the student behavior, and emotional and cognitive engagement progress?
4. Describe your experience of doing online teaching through the PBOL method using Microsoft Team and social media in entrepreneurship class?
5. When you apply the entrepreneurship teaching by using the PBOL method, explain how your students’ social behavior, feelings, and thinking skills are in discussing in class?
6. When you actively apply the PBOL method in the entrepreneurship class, explain what kind of academic work students enjoy with it?
7. In one class period, how many times do students engage actively in the virtual discussion via Microsoft Team and other supporting social media?
8. Give your opinion about the contribution of the PBOL method to increasing student engagement and learning outcomes?
9. Describe the effectiveness of your teaching an entrepreneurial project task through the Microsoft Team-based PBOL method and social media?.
10. Describe the type of academic work you believe will interest your student and help them independently to learn at home by using PBOL?
11. List some of how a PBOL Metod can help students engage in entrepreneurship learning and improve their learning outcomes?

Closing remarks:

Thank you, ___________ (lecturer) for your participation in this interview. Once your responses have been transcribed, I will provide you with a transcript of this interview after the observation and after the research results are published. Over the next few weeks, I will be watching you apply the PBOL lessons in your entrepreneurship class at the Faculty of Education, State University of Padang. Thank you again for your participation in this research.

**Student Interview Protocol**

**Project:**          The Effect of Project-Based Online Learning Methods (PBOL) and Student Engagement on Entrepreneurship Learning Outcomes

Interview Time :

Date :

Place : Padang State University

Interviewer : Drs. Zelhendri Zen, M.Pd. PhD

Interviewee :

Resource Person Position: Entrepreneurship Student

Thank you for taking the time to talk to me about your perception of online lectures in entrepreneurship classes at the Padang State University Faculty of Education students.

You were selected to participate because you are currently a student in entrepreneurship courses at the Faculty of Education, State University of Padang. To improve the results and quality of student learning in entrepreneurship courses, this research study is designed to provide a platform so that your voice, opinion, and input on the impact of applying the PBOL method and student engagement on student learning outcomes that you experience in your entrepreneurship class can be heard. revealed.

Please note that there are no right or wrong answers to these questions. My role is simply to record information and share information that offers a glimpse of your experience in an entrepreneurship class.

Please note that all of your responses will remain confidential, and identifying information will be removed from the transcript. You may withdraw from this interview at any time without penalty. Your responses to these interview questions will form part of my research into the adoption of the PBOL method and student engagement in learning outcomes in entrepreneurship classes.

Our interview today should take no more than 30 minutes. As you'll notice, I rigorously recorded audio of our session to help me transcribe and analyze your feedback. After the interview is transcribed, I will provide a transcript, or a detailed document that includes your responses, so you can verify its accuracy.

[Confirm that I have received the student consent form ahead of the interview.]

[Turn on the digital recorder and test it.]

_____________ (student), do you have any questions before we start?

Initial Student Interview Questions:

Introduction: Let’s begin with some background information on what it means to be engaged. Being “engaged” considers three forms: behavioral/social engagement, emotional engagement, and cognitive engagement. Behavioral/social engagement includes how you demonstrate “the behaviors expected in a classroom— such as listening, doing assignments, following directions, participating, and so on” (Cooper, 2014, p. 365). Additionally, emotional engagement includes your sense of positivity for a class, your feelings of being satisfied, comfortable and interested, and your desire to find success in mathematics class (Blumenfeld et al., 2006; J. A. Fredricks et al., 2004). And finally, cognitive engagement includes the amount of mental energy you exert, the ways that you are thinking about entrepreneurship content, and the ways you investigate new information and work with mental challenges (Cooper, 2014).

By understanding student engagement, explain what it means when a student is "engaged" in their learning in an entrepreneurship class by using Project Based Online Learning Method.

1. Describe your behavior, feelings, and impressions during the PBOL method of learning with the Microsoft Team and social media in the entrepreneurship class?
2. As a student when you are actively involved in learning in an entrepreneurship class using the PBOL method, how is your cognitive progress in understanding the material?
3. Describe the experience of doing independent learning through the PBOL method using Microsoft Team and social media in entrepreneurship class?
4. When you take entrepreneurship learning using the PBOL method, explain how your social behavior, feelings, and thinking skills are in discussing in class?
5. When you feel you are actively involved in following the PBOL method in the entrepreneurship class, explain what kind of academic work you enjoy doing?
6. In one class period, how many times did you ask questions in the virtual class via Microsoft Team and other supporting social media and how many times did you contribute during the discussion during synchronous and asynchronous classes?
7. Describe your behavior when asked to complete an entrepreneurial project task through the Microsoft Team-based PBOL method and social media?.
8. Describe the type of academic work you believe will interest you and help you study independently at home?
9. List some of how a lecturer can help you engage in entrepreneurship learning!
10. Explain in your opinion how do lecturers carry out PBOL learning based on Microsoft Team in entrepreneurship class? Do you find it interesting?" and explain how the lecturer measures your level of learning achievement using this PBOL method?

Closing remarks:

Thank you, ___________ (student) for your participation in this interview. Once your responses have been transcribed, I will provide you with a transcript of this interview after the observation and after the research results are published. Over the next few weeks, I will be watching you participate in PBOL lessons in your entrepreneurship class at the Faculty of Education, State University of Padang. Thank you again for your participation in this research.

**Appendix 2**

**QUESTIONNAIRE**

**The Effect of Project-Based Online Learning Methods (PBOL) and Student Engagement on Entrepreneurship Learning Outcomes**

**PART A : STUDENT DATA**

Please write or tick “√” in the box based on the following questions:

1. Age: ( ) 18-20 years old

( ) 20-22 years old

( ) > 22 years old

2. Gender: ( ) Male

( ) Female

Institution :.................................................

Department :.................................................

**PART B: STUDENT ENGAGEMENT QUESTIONNAIRE**

This questionnaire consists of 20 statement items, aims to measure students' engagement in following entrepreneurship class, fill out the entire questionnaire according to the instructions below. What you fill in has nothing to do with your grades, therefore, fill out each statement item honestly according to what you experienced, felt, and did after attending lectures based on PBOL methods. Make sure you have filled out all the statements in this questionnaire.

**Directions**: This questionnaire evaluate your engagement and perceived learning in online environments based on the following project-based online learning course consisting of behavioral engagement, emotional engagement, and cognitive engagement during student perceived learning interaction. Read each statement and use the associated Likert scale to select which best reflects your opinion. Scale: 1 = Never (N), 2 = Rarely (R) 3 = Sometimes (S), 4 = Very Often (VO), 5 = Always (A).

| **NO** | **ITEM** | **Never (N)** | **Rarely (R)** | **Sometimes (S)** | **Very Often (VO)** | **Always (A)** |
| --- | --- | --- | --- | --- | --- | --- |
|  |  | **1** | **2** | **3** | **4** | **5** |
| **Behavioral Engagement** | |  |  |  |  |  |
| 1 | I actively answer the lecturer's questions, express new ideas, and enjoy discussions and debates during online learning. | **1** | **2** | **3** | **4** | **5** |
| 2 | I actively observe, memorize, and follow the stages of building a business project through online learning. | **1** | **2** | **3** | **4** | **5** |
| 3 | I actively think and ask questions about things I don't understand in the virtual class and on social media. | **1** | **2** | **3** | **4** | **5** |
| 4 | I actively think associative, comparing the difficulties I get while working on the project using PBOL. | **1** | **2** | **3** | **4** | **5** |
| 5 | I am actively improving my knowledge and skills related to working on business projects through the PBOL method. | **1** | **2** | **3** | **4** | **5** |
| 6 | I diligently read to get information about business building tips and strategies independently through PBOL using Microsoft teams and social media. | **1** | **2** | **3** | **4** | **5** |
| 7 | I am actively asking about business feasibility calculations that I need to master through PBOL using Microsoft teams and social media. | **1** | **2** | **3** | **4** | **5** |
| 8 | I am more actively practicing doing business feasibility calculations in following the entrepreneurship subject through PBOL using Microsoft teams and social media. | **1** | **2** | **3** | **4** | **5** |
| **Emotional Engagement** | |  |  |  |  |  |
| 9 | I like entrepreneurship college through PBOL using Microsoft teams and social media because it can give me the skills to be a new entrepreneur. | **1** | **2** | **3** | **4** | **5** |
| 10 | I like working on projects assigned by the lecturer under PBOL using Microsoft teams and social media. | **1** | **2** | **3** | **4** | **5** |
| 11 | I like to work together in groups doing project assignments assigned by the lecturer through PBOL using Microsoft teams and social media. | **1** | **2** | **3** | **4** | **5** |
| 12 | I like to learn to understand business-building strategies through video tutorials under PBOL using Microsoft teams and social media. | **1** | **2** | **3** | **4** | **5** |
| 13 | I am happy to take entrepreneurship courses because they can improve my skills to become a prospective new entrepreneur, even learning through PBOL by using Microsoft teams and social media is fun. | **1** | **2** | **3** | **4** | **5** |
| 14 | I'm excited to join this project-based entrepreneurship online class. | **1** | **2** | **3** | **4** | **5** |
| **Cognitive Engagement** | |  |  |  |  |  |
| 15 | I am preparing to build a digital business through project-based lectures. | **1** | **2** | **3** | **4** | **5** |
| 16 | I concentrate and actively listen to the material in class through PBOL using Microsoft teams and social media. | **1** | **2** | **3** | **4** | **5** |
| 17 | I am enthusiastic about participating in all entrepreneurship lecture activities, both in the online method and offline. | **1** | **2** | **3** | **4** | **5** |
| 18 | I am determined to be able to master the science of business and want to be a successful entrepreneur through the PBOL method. | **1** | **2** | **3** | **4** | **5** |
| 19 | I studied hard and tried to build a small business in the form of a college project through PBOL by using Microsoft teams and social media. | **1** | **2** | **3** | **4** | **5** |
| 20 | I attend every entrepreneurship lecture through the PBOL method using Microsoft teams and social media on time, both in virtual synchronous and asynchronous. | **1** | **2** | **3** | **4** | **5** |

**Appendix 3**

**PORTFOLIO ASSESSMENT SHEET**

Expertise Package : Creative Industry Business

Subject : Entrepreneurship

Time Allocation : One Semester

Student's Name :

| NO | BASIC SKILLS COMPETENCIES | CRITERIA | | | | SCORE | CATEGORY* |
| --- | --- | --- | --- | --- | --- | --- | --- |
|  |  | SKILLS | COMPATIBILITY | PROBLEM SOLVING | QUALITY |  |  |
| CONCEPT MASTERY | |  |  |  |  |  |  |
| 1 | Remembering |  |  |  |  |  |  |
| 2 | Understanding |  |  |  |  |  |  |
| 3 | Applying |  |  |  |  |  |  |
| 4 | Analyzing |  |  |  |  |  |  |
| 5 | Evaluating |  |  |  |  |  |  |
| 6 | Creating |  |  |  |  |  |  |
| AVERAGE FOR CONCEPT MASTERY | | | | | |  |  |
| CREATIVITY | |  |  |  |  |  |  |
| 1 | Curiosity |  |  |  |  |  |  |
| 2 | Asking a lot |  |  |  |  |  |  |
| 3 | Lots of ideas |  |  |  |  |  |  |
| AVERAGE SCORE FOR CREATIVITY | | | | | |  |  |
| LEADERSHIP | |  |  |  |  |  |  |
| 1 | Analyze and make decisions. |  |  |  |  |  |  |
| 2 | Motivating |  |  |  |  |  |  |
| 3 | Communication |  |  |  |  |  |  |
| 4 | Responsibility |  |  |  |  |  |  |
| AVERAGE SCORE FOR LEADERSHIP | | | | | |  |  |
| COURAGE TO TAKE RISKS | |  |  |  |  |  |  |
| 1 | Dare to speculate |  |  |  |  |  |  |
| 2 | Not afraid to fail |  |  |  |  |  |  |
| 3 | High confidence |  |  |  |  |  |  |
| AVERAGE SCORE FOR COURAGE TO TAKE RISKS | | | | | |  |  |
| ENTREPRENEURSHIP ENCOURAGEMENT | |  |  |  |  |  |  |
| 1 | Interest and Motivation |  |  |  |  |  |  |
| 2 | Optimism |  |  |  |  |  |  |
| 3 | Self-confidence |  |  |  |  |  |  |
| AVERAGE SCORE FOR ENTREPRENEURSHIP ENCOURAGEMENT | | | | | |  |  |

*Category: 0-50 (Poor), 51-79 (Good) and 80-100 (Excellent)
